# Independent influence of negative blood cultures and bloodstream infections on in-hospital mortality

**DOI:** 10.1186/1471-2334-14-36

**Published:** 2014-01-21

**Authors:** Carl van Walraven, Jenna Wong

**Affiliations:** 1 Faculty of Medicine, University of Ottawa, 451 Smyth Rd, Ottawa, Ontario, Canada; 2 McGill University, Montréal, Canada; 3 Ottawa Hospital Research Institute, Clinical Epidemiology Program, ASB-1, 1053 Carling Ave, Ottawa, Ontario K1Y 4E9, Canada; 4 ICES@uOttawa, ASB-1, 1053 Carling Ave, Ottawa, Ontario, Canada

**Keywords:** Blood stream infections, Hospital mortality, Outcomes, Proportional hazards modeling

## Abstract

**Background:**

The independent influence of blood culture testing and bloodstream infection (BSI) on hospital mortality is unclear.

**Methods:**

We included all adults treated in non-psychiatric services at our hospital between 2004 and 2011. We identified all blood cultures and their results to determine the independent association of blood culture testing and BSI on death in hospital using proportional hazards modeling that adjusted for important covariates.

**Results:**

Of 297 070 hospitalizations, 48 423 had negative blood cultures and 5274 had BSI. 12 529 (4.2%) died in hospital. Compared to those without blood cultures, culture-negative patients and those with BSI were sicker. Culture-negative patients had a significantly increased risk of death in hospital (adjusted hazard ratio [HR] ranging between 3.1 and 4.4 depending on admission urgency, extent of comorbidities, and whether the blood culture was taken in the intensive care unit). Patients with BSI had a significantly increased risk of death (adj-HR ranging between 3.8 and 24.3] that was significantly higher when BSI was: diagnosed within the first hospital day; polymicrobial; in patients who were exposed to immunosuppressants or were neutropenic; or due to *Clostridial* and *Candidal* organisms. Death risk in culture negative and bloodstream infection patients decreased significantly with time.

**Conclusions:**

Risk of death in hospital is independently increased both in patients with negative blood cultures and further in those with bloodstream infection. Death risk associated with bloodstream infections varied by the patient’s immune status and the causative microorganism.

## Background

Blood cultures are commonly ordered in clinical practice with bloodstream infections found in almost all medical specialties. Blood cultures are typically ordered when physicians suspect the possibility of a bloodstream infection. Both the test and its result signal important events in a patient’s care: blood cultures are often performed when patients are ill or when their conditions significantly worsen; bloodstream infections frequently change treatment, often invoke a search for both causes and manifestations of the infection, and may indicate a significant turning point in a patient’s health.

Despite the prevalence of blood cultures and the importance of bloodstream infections, their impact on hospitalized patient outcomes is unclear. Previous studies of blood cultures and hospital mortality have focused on specific patient populations [1-6], specific microorganisms [7-10], or both [11]. Other studies focused their mortality analysis on patients with documented bloodstream infections to the exclusion of those with negative blood cultures [12-16]. A few studies have compared hospital mortality in patients with or without a bloodstream infection but studied a restricted population including those admitted with acute on chronic hepatic failure [1] and critically ill patients with catheter-related blood stream infections [2]. Finally, no previous studies have determined the influence of simply having a blood culture measured during the hospital – independent of its results – on hospital mortality in a broad patient population.

Knowing the effect of bloodstream infections on patient outcomes is needed to determine their importance in patient care. In this study, we measured the independent influence of blood cultures and bloodstream infections on the risk of death in hospital.

## Methods

### Study setting

This study took place at The Ottawa Hospital (TOH), a tertiary-care teaching facility with three in-patient sites that averaged 20 000 admissions annually during the study period. TOH functions within a publicly funded health care system, is the sole trauma centre for the region, and provides most of the region’s oncological, thoracic surgery, and neurosurgical care.

### Patients

We included all non-psychiatric hospitalizations of people over 15 years of age between 1 April 2004 and 31 March 2011 including same-day surgeries. This study period was chosen to maximize the study sample size given the data available at the time we conducted the study. Psychiatry admissions were not included since physically ill patients potentially requiring blood cultures are rarely admitted to – or are readily transferred from – the psychiatry service. We excluded patients who were transferred from or to other hospitals since we could not get admission data or mortality status, respectively. The unit of analysis in this study was the hospitalization.

### Blood culture utilization and results

We linked to the laboratory database to determine if and when each hospitalized patient had a blood culture measured. Cultures collected in the emergency department prior to admission were also captured and attributed to the hospitalization. Each day of each person’s hospitalization was divided into six-hour sections starting at the time the patient was admitted to hospital. For each of these quarter-day segments, we determined whether or not the person had at least one blood culture measured.

Results of all blood cultures were then retrieved. Likely contaminants were identified using criteria modified from Richter [17] in which coagulase-negative staphylococci, aerobic and anaerobic diphtheroids, *Micrococcus* spp., *Bacillus* spp., and viridans group streptococci were classified as contaminants in the absence of another blood culture within 48 hours growing the same organism. Microorganisms identified on the final report were clustered by their genus into microorganism groups that were based on the chapter (in a commonly used textbook of infectious diseases [18]) which contained most information about the organism. Blood cultures growing more than one organism were classified as polymicrobial.

### Outcome and covariates

The primary outcome of the study was all-cause death in hospital. The primary covariate was the risk of death in hospital calculated using an extension of a model by Escobar *et al.*[19]. The “Escobar model” estimates the probability of death in hospital based on covariates available at the time of admission including: patient age and sex; admission urgency (i.e. elective or emergent) and service (i.e. medical or surgical); admission diagnosis; severity of acute illness as measured by the Laboratory-based Acute Physiology Score (LAPS); chronic comorbidities as measured by the Elixhauser score [20]; and admission diagnosis [19]. The Escobar model was highly discriminative, well calibrated, and was externally validated in our center with a c-statistic of 0.901 [21].

In the present study, we used a validated extension of the Escobar model to calculate each person’s *daily* risk of death in hospital [22]. The “Daily Escobar” model included all of the covariates in the Escobar model but expressed LAPS as a time-dependent covariate (i.e. its value was allowed to change with time) in which we used the most extreme value of each laboratory test in each 6-hour segment to calculate the LAPS. It also included three additional time-dependent covariates: admission to intensive care unit [determined by linking to the hospital’s patient location table]; performance of significant operative procedures [23] [determined by linking to the hospital’s procedure table]; and awaiting long-term care status. The “Daily Escobar” model also had excellent discrimination (concordance probability of 0.895, 95% CI 0.889-0.902) and calibration.

Other time-dependent covariates were also examined. We linked to the hospital’s laboratory dataset to determine each patient’s neutrophil count throughout the admission; counts <500 × 10^9^ neutrophils/mL were classified as neutropenic. We also linked to the pharmacy dataset to determine if patients received immunosuppressive medications including enteral or parenteral steroids, methotrexate, azathioprine, mycophenolate mofetil, cyclosporine, or anti-thymocyte globulin.

### Analysis

For baseline descriptive purposes, we separated patients into three exclusive groups: no blood cultures done during hospitalization; at least one blood culture but no bloodstream infection; or one or more bloodstream infections. The prevalence - or median value - of each covariate within each group was described. Blood culture utilization was expressed as an incidence density reported as the number of tests per 100 patient-days.

We described how blood culture utilization and bloodstream infections changed hospital mortality by calculating the *daily expected number of deaths* in each patient group. For all patients in each group, we calculated the estimated risk of death using the ‘Daily Escobar’ model and then summed these daily risks to determine the expected number of deaths in each group on each day. In this analysis, patients with a blood culture had their reference time (i.e. time 0) set at the date of their first blood culture. Patients with no blood cultures had their reference time set at the middle of their admission.

We then determined the association of blood culture results with time to death in hospital using a survival model. Observation started at hospital admission and was censored at discharge with observation time broken into six hour segments. Blood culture procurement and bloodstream infection status was expressed as time-dependent binomial covariates. We added to the model a term expressing the number of quarters since the blood culture had been done to quantify changes over time between the association of blood culture measurement and death in hospital. The best fitting polynomial transformation that captured this change was determined using a modification of a SAS macro from Sauerbrei [24]. We included terms capturing polymicrobial status and timing of bloodstream infection (categorized as admission [positive blood culture procured within first day of admission] vs. hospital [blood culture procured more than 24 hours after admission]). We also determined if significant interactions (p < 0.001) existed between each covariate and blood culture measurement or bloodstream infections.

**Figure 1:**
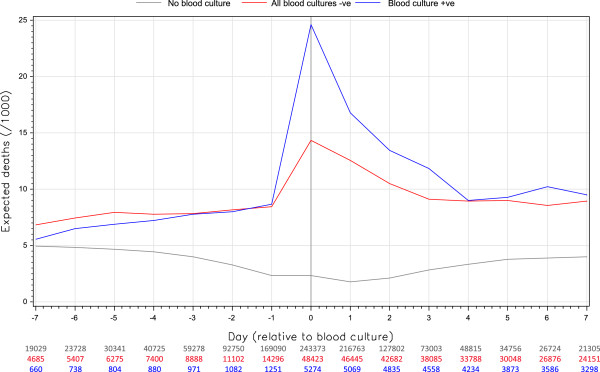
**Daily expected risk of death by blood culture status.** The daily hazard of death was calculated for each person using a validated predictive model that captured daily values of important, patient-level covariates [22]. Within each group (no blood culture [red], negative blood culture [grey], positive blood culture [blue]), these were summed and standardized to 1000 population (vertical axis). The horizontal axis displays the hospital day relative to the first blood culture; for patients with no blood culture, the hospitalization midpoint was used as the reference. The dip in the ‘no blood culture’ group is due to increased prevalence at zero time of short stay admissions (which have the lowest expected risk of death).

The fully adjusted model determining the association of blood cultures and bloodstream infections with death in hospital controlled for each patient’s daily expected risk of death in hospital. This was quantified using the “Daily Escobar” model described above to calculate the ***Xβ*** from the model for each patient on each day. This daily death risk was expressed as a time-dependent covariate but was kept constant after patients were diagnosed with a bloodstream infection. This was done because the inclusion of the daily death risk in the model – including that after bloodstream infection was treated – would mute the influence of bloodstream infection on outcomes. This is because the *treatment* of bloodstream infection should decrease the daily death risk score (see Figure 1) and lower daily death risk scores are strongly associated with a decreased risk of death. Therefore, including in the model post-treatment daily death risk scores of bloodstream infection patients would, because of confounding, bias the association between bloodstream infection and hospital death towards the null. Fisher highlighted this potential problem with survival models using time-dependent covariates [25].

Other time-dependent covariates offered to the model included those capturing each patient’s neutropenic status and exposure to immunosuppressant medications. To determine if death risk varied by microorganism, we subclassified bloodstream infection patients by the microorganism group of the isolate(s). Also, we repeated the model using only one admission per patient (i.e. a patient-level analysis rather than an admission level analysis) to determine if our conclusions were sensitive to this modification. Finally, we determined the possible influence of censoring patients at hospital discharge by conducting a competing risks model using the methods described by Wolkewitz et al. {13665}. This was accomplished by assessing the size of parameter estimates in a replicate of our final model that had time to discharge from hospital as the outcome while censoring patients who died during their admission. The study was approved by The Ottawa Hospital Research Ethics Board.

## Results

During the study period, a total of 317 194 adult in-patient encounters (in which patients were neither transferred from or to another facility) occurred at our hospital. 20 124 of these hospitalizations were excluded because patients were admitted and discharged from the psychiatry service.

This left a total of 297 070 hospitalizations consisting of 186 182 patients (Table 1). Most hospitalizations had no blood cultures (n = 243 373, 81.9%), 16.3% of admissions (N = 48 423) had all negative cultures, and 1.8% (N = 5274) had at least one documented bloodstream infection. Regardless of the result, patients having one or more blood cultures during the hospitalization were notably sicker than those without (Table 1): they were older; they had higher Elixhauser [20] scores (indicating more extensive chronic illnesses); they were more likely to be admitted urgently and had a much higher predicted risk of death in hospital; they had more extensive perturbations of their laboratory tests (as indicated by their LAP Scores); they were more likely to be treated in the intensive care unit or to be exposed to immunosuppressants at any time during the admission; and they had a longer median length of stay.

**Table 1:** Description of study hospitalizations

	**No blood culture (N=243 373, 81.9%)**		**1+ Blood culture, all negative (N=48 423, 16.3%)**		**1+ Blood culture, 1+ positive (N=5274, 1.8%)**		**All patients (N=297, 7%)**	
***Patient***								
Mean age (SD)	53.2	20.2	62.3	18.8	63.4	17.9	54.8	20.2
Female	145 772	59.9	23 414	48.4	2403	45.6	171 589	57.8
Elixhauser score*: <0	11206	4.6	1719	3.6	212	4.0	13 137	4.4
0	140 027	57.5	12 461	25.7	1177	22.3	153 665	51.7
>0	91 140	37.9	34 243	70.7	3885	73.7	130 268	43.8
***Admission***								
Emergent admission	135 962	55.9	43 914	90.7	4856	92.1	184 732	62.2
Admitted to surgical service	82 721	34.0	11 116	23.0	1012	19.2	94 849	31.9
Median death risk (IQR)**	0.4%	0.1-2.1	5.1%	1.2-15.6	8.0%	2.1-22.6	0.6%	0.17-4.0
LAP score****: 0	152 338	62.6	9742	20.1	804	15.2	162 884	54.8
>0	91 035	37.4	38 681	79.9	4470	84.8	134 186	45.2
***Hospitalization***								
Intensive care unit***	4137	1.7	7845	16.2	1297	24.5	13 369	4.5
Surgical procedure***	66 928	27.5	8813	18.2	964	18.2	76 649	25.8
Neutrophils < 500 × 10^9^/L***	730	0.3	3147	6.5	609	11.5	4456	1.5
Immunosupressant***	6814	2.8	14 140	29.2	1763	33.3	22 579	7.6
Awaiting placement anytime	97	0.04	3680	7.6	434	8.2	4159	1.4
Median LOS (IQR)	4	2-6	9	4-19	12	6-28	4	2-8
Died in hospital	5013	2.1	6423	13.3	1093	20.7	12 529	4.2

Unless stated, numbers in right column are percentages.

*SD* standard deviation, *IQR* interquartile range.

*Measures number and severity of comorbidities [21].

**Measured using Escobar model [20] using covariate values at hospital admission.

***At any time during the hospitalization.

****Laboratory-based Acute Physiology Score; quantifies deviations of important laboratory tests from normal [20].

### Blood culture utilization and results

The incident rate for blood culture utilization was 3.7 culture sets per 100 patient days; bloodstream infection incident rate was 0.25 per 100 patient days. Blood culture utilization was heavily weighted to the start of the hospital stay; blood culture utilization rates were highest on the first hospital day (4.5 cultures per 100 patient-days) while utilization was subsequently significantly lower (an average of 0.68 cultures per 100 patient-days [range 0.55-0.79]). Bloodstream infection rates were also highest on the first hospitalization day (0.41 bloodstream infections per 100 patient-days) and decreased significantly for the rest of the hospitalization (mean rate of 0.03 bloodstream infections per 100 patient-days, range 0.02-0.05).

Table 2 shows that the majority of people undergoing testing had only one set of blood cultures (37 243 people, 69.4% of those having any blood culture). As well, 4721 of the 5274 people (89.5%) with at least one positive test had only one bloodstream infection. The ten most common microorganisms identified in the positive blood cultures are shown in Table 3 with *Enterobacteriacae, S. Aureus,* and *Streptococci* being the most common. 637 of a total of 7549 bloodstream infections (8.4%) were polymicrobial with one or more polymicrobial bloodstream infections occurring in 502 (0.2%) hospitalizations.

**Table 2:** Summary of blood culture utilization and results

		**Number of blood culture sets**				
		**0**	**1**	**2**	**3+**	**Total**
**NUMBER OF POSITIVE CULTURES**	**0**	**243 373**	**34 854**	**8023**	**5546**	291 796 (98.2%)
	**1**	-	**2389**	**1101**	**1231**	4721 (1.6%)
	**2**	-	-	**107**	**317**	424 (0.1%)
	**3+**	-	-	-	**129**	129 (0.04%)
	**TOTAL**	243 373	37 243	9231	7223	297 070
		(81.9%)	(12.5%)	(3.1%)	(2.4%)	

A maximum of 1 culture set per person per six hour period was counted.

**Table 3:** Description of 8334 microoganisms identified in 7549 positive cultures

**Microorganism group**	**Isolates**	**Frequency**	**% Total (% of group)**
Enterbacteriacae	*-*	3024	36.3
	*Escherichia coli*	1659	(54.9)
	*Klebsiella pneumoniae*	534	(17.7)
	*Enterobacter cloacae*	221	(7.3)
	Other	610	(20.2)
*Staphylococcus Aureus*	*-*	1065	12.8
Streptococci	*-*	979	11.7
	*Streptococcus pneumoniae*	321	(32.8)
	Group B *Streptococcus* (*s. agalactiae*)	131	(13.4)
	Viridans group *Streptococcus*	105	(10.7)
	Other	422	(43.1)
Enterococcus (including Streptococcus bovis)	*-*	689	8.3
	*Enterococcus faecalis*	412	(59.8)
	*Enterococcus faecium*	211	(30.6)
	Enterococcus species	31	(4.5)
	Other	35	(5.1)
Candida	*-*	574	6.9
	*Candida albicans*	292	(50.9)
	*Candida (torulopsis) glabrata*	119	(20.7)
	*Candida parapsilosis*	65	(11.3)
	Other	98	(17.1)
Other gram negative and gram-variable bacilli	*-*	538	6.5
	*Pseudomonas aeruginosa*	381	(70.8)
	Gram-negative bacilli	69	(12.8)
	*Achromobacter (alcaligenes) xylosoxidans*	17	(3.2)
	Other	71	(13.2)
Anaerobic gram-positive nonsporulating bacilli	*-*	239	2.9
	*Propionibacterium acnes*	114	(47.7)
	Propionibacterium species	102	(42.7)
	*Eubacterium lentum*	16	(6.7)
	Other	7	(2.9)
Other anaerobes	*-*	160	1.9
	*Bacteroides fragilis*	99	(61.9)
	*Bacteroides thetaiotaomicron (fragilis gr.)*	13	(8.1)
	*Fusobacterium nucleatum*	10	(6.3)
	Other	38	(23.8)
Methicillin resistant *staphylococcus aureus*	*-*	153	1.8
Clostridium	*-*	116	1.4
	*Clostridium perfringens*	49	(42.2)
	*Clostridium septicum*	23	(19.8)
	Clostridium species	18	(15.5)
	Other	26	(22.4)
Other	*-*	797	9.6
	Coagulase-negative *Staphylococcus*	158	(19.8)
	*Stenotrophomonas maltophilia*	70	(8.8)
	*Haemophilus influenzae*	52	(6.5)
	Other	517	(64.9)

Blood cultures growing two organisms in the same class were counted once. Microorganisms were classified using Mandell [19].

### Blood cultures, bloodstream infections and unadjusted risk of hospital mortality

Patients without blood cultures were significantly less likely to die in hospital (2.1%) than those with all negative blood cultures (13.3%) or those with at least one bloodstream infection (20.7%) (Table 1; χ^2^ value 16172, p < 0.0001).

Expected death risk varied by patient group. In patients without blood cultures, the daily expected risk of death averaged 3.5 deaths per 1000 patient-days (range 1.8-5.0); the lowest expected death rates for this group occurred at Time 0 (Figure 1) likely due to an increased prevalence at this point of short stay admissions which have the lowest expected risk of death. In contrast, the daily expected risk of death in patients with blood cultures - regardless of their result - averaged 7.5 deaths per 1000 (range 5.6-8.7) prior to the blood culture testing (Figure 1). On the day of the blood culture, however, expected death rates increased significantly to 14.3 deaths per 1000 patient-days in patients with negative cultures and 24.6 deaths per 1000 patient-days in patients with bloodstream infections. Expected death rates returned to pre-testing values within 4 days of blood culture procurement (Figure 1).

**Figure 2:**
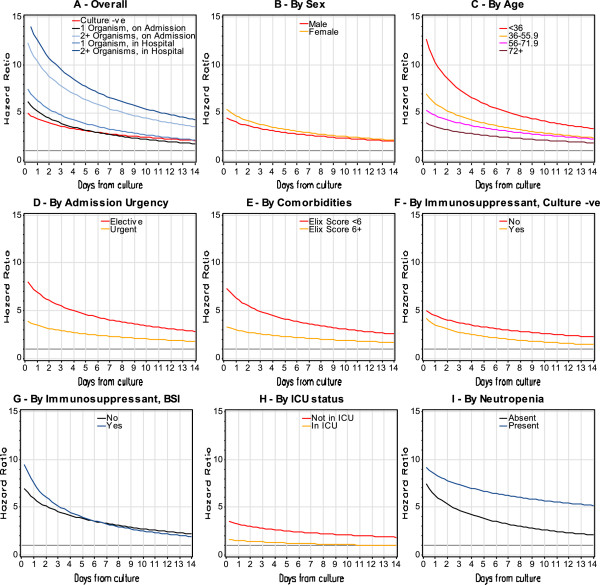
**Stratified and unadjusted influence of negative blood cultures and bloodstream infection on risk of death in hospital.** These figures plot the association between day from blood culture (horizontal axis) with relative hazard of all-cause death in hospital (vertical axis) for patients with negative blood culture (red lines) and bloodstream infection (blue lines). These estimates were generated from models in which blood cultures were expressed as time-dependent covariates and are stratified by the characteristic in each title; therefore, the displayed hazard of death is relative to patients in that strata who did not have blood culture testing. Plot **A** shows the unadjusted association. In the remaining plots **(Plot B through I)**, the stratifying variable for the analysis is presented atop the plot. The p-value for all of the interactions presented here (i.e. plots **B** to **F**) is ≤ 0.0001.

Both negative blood cultures and bloodstream infection were associated with an increased risk of death in hospital (Figure 2A). The unadjusted death risk in patients with negative blood cultures was five times that of people without blood cultures. The unadjusted hazard ratio for death in patients with bloodstream infection ranged between 6.2 and 15.8 depending on both the timing of the bloodstream infection (i.e. at admission vs. in-hospital) and the number of organisms in the positive culture: patients with bloodstream infection detected *after* the first hospitalization day had a significantly higher *unadjusted* risk of death compared to bloodstream infections detected at admission; unadjusted death risk was higher in polymicrobial bloodstream infections than in single-microbial bloodstream infections (Figure 2A). The increased risk of death associated with negative blood cultures and bloodstream infection both decreased significantly over time.

The unadjusted relative hazard of death in hospital with negative blood cultures and bloodstream infection varied significantly with several covariates. Compared to a patient without blood culture testing, the unadjusted relative risk of death associated with negative blood cultures was significantly higher in patient groups having a lower risk of death including: females (Figure 2B); younger patients (Figure 2C); those admitted electively to hospital (Figure 2D); patients with fewer comorbidities (Figure 2E); those without immunosuppressants (Figure 2G); and patients *not* in the intensive care unit (Figure 2H). The increased risk of death associated with bloodstream infection interacted significantly with neutropenia (Figure 2I) and immunosuppressant exposure (Figures 2F and 2G); compared to patients without blood culture testing, the risk of death with bloodstream infection was extensively and significantly higher in patients who were neutropenic and those exposed to immunosuppressants.

### Adjusted association of blood cultures and, bloodstream infections with hospital mortality

**Figure 3:**
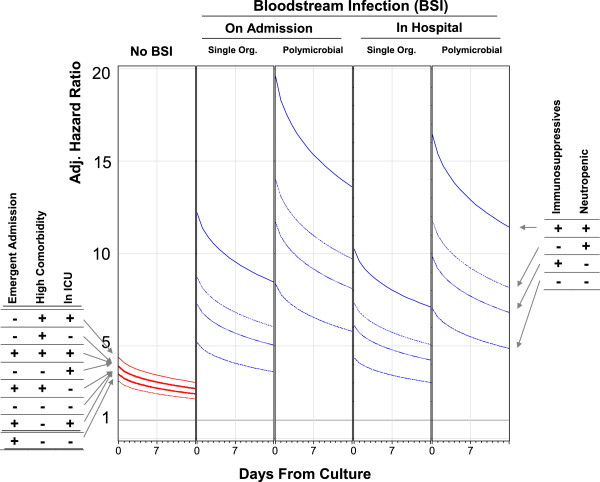
**Adjusted influence of negative blood cultures/bloodstream infection on hospital mortality by significant effect modifiers.** These plots present the adjusted hazard ratio (vertical axes) for the association of negative blood cultures (“No BSI”) and bloodstream infection with death in hospital in the first 2 weeks following blood culture testing (horizontal axes). Adjusted hazard ratios for negative blood cultures are presented for all combinations of admission urgency (elective vs. emergent), patient comorbidity status (low comorbidity [Elixhauser score of 1] vs. high comorbidity [Elixhauser score of 12]), and intensive care unit (ICU) status. Adjusted hazard ratios for bloodstream infection (calculated for patients with low comorbidity, low comorbidity, and not in the ICU) are presented for all combinations of immunosuppressant exposure and neutropenia. All hazard ratios adjust for: patient age; patient sex; admission service (i.e. medical or surgical) and diagnosis; severity of acute illness as measured by the Laboratory-based Acute Physiology Score (LAPS); chronic comorbidities as measured by the Elixhauser score [20]; treatment in the intensive care unit; performance of significant operative procedures [23]; awaiting long-term care status; [19] and exposure to immunosuppressant medications. All adjusted hazard ratios use as a comparator a person an electively admitted person with low comorbidity not in the ICU who has no blood culture measured.

After adjusting for important covariates, the risk of death in hospital was significantly higher in patients with negative blood cultures (Figure 3A, Additional file 1). This risk ranged between 3.1 and 4.4 times that of people without blood culture testing, varying significantly by admission urgency (risk tended to be higher in elective admissions), patient comorbidity (risk tended to be higher in sicker patients), and ICU status (risk tended to be higher in ICU patients). The increased risk of death associated with negative blood cultures decreased significantly over time.

Bloodstream infections conferred a significant, additional adjusted risk of death in hospital beyond that from blood culture testing (Figure 3B-E, Additional file 1). This risk was higher in bloodstream infections identified in the first 24 hours of the admission and in those due to multiple microorganisms (Figure 3B vs. 3D and Figure 3C vs. 3E). In addition, the adjusted risk of death associated with bloodstream infections increased significantly in patients exposed to immunosuppressives, those who were neutropenic, or both. On the first day of bloodstream infection, the adjusted relative hazard of death in hospital varied from a low of 3.8 [95% CI 3.1-4.6] (in: emergently admitted patients; with low comorbidity; not in the ICU; without neutropenia; not on immunosuppresives; with a bloodstream infection from a single organism during the hospitalization) to 24.3 [95% CI 16.6-35.4] (in: electively admitted patients; with high comorbidity; in the ICU; with neutropenia; on immunosuppresives; with a polymicrobial bloodstream infection identified in the first 24 hours of the hospitalization). As with negative blood cultures, the increased risk of death from bloodstream infections decreased significantly over time (Figure 3B- 3E).

**Figure 4:**
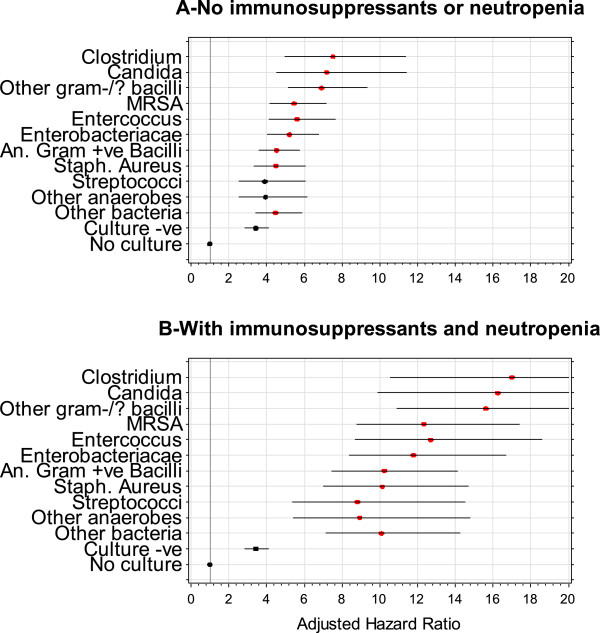
**Independent association of specific microorganism classes with hospital death risk.** These plots present the adjusted hazard ratio of death in hospital (horizontal axis) for patients without blood culture testing, those with negative blood cultures, and those with bloodstream infections caused by different microorganisms (vertical axis). Estimates are provided with 95% confidence intervals and are presented for patients without immunosuppressive or neutropenia **(Plot A)** and for patients with immunosuppressive or neutropenia **(Plot B)**. In both plots, adjusted hazard ratios are relative to people without blood culture testing. Microorganisms whose adjusted relative hazard is statistically distinct from patients with culture negative blood cultures are indicated in red.

The influence of bloodstream infection on the independent risk of death in hospital varied significantly by the microorganism isolated in the culture (Figure 4). Bloodstream infections with *Clostridial, Candida,* and other gram negative/variable bacilli had adjusted death risks that were notably higher than the other microorganisms. Figure 4 also highlights the striking increased risk of death in hospital from bloodstream infection when patients were neutropenic and exposed to immunosupressives.

The study’s conclusions did not change significantly when we repeated the analysis with the patient – rather than the admission – as the unit of analysis (Additional file 2). All but one of the parameter estimates in the patient model - that for polymicrobial status - remained within the 95% confidence intervals of the hospitalization model (Additional file 1). Changes in the influence of polymicrobial status on mortality might be due to a change in statistical power (i.e. a reduction in the number of polymicrobial cases when the unit of analysis changed to the patient) or might indicate that *repeated* polymicrobial bloodstream infections (which would be in the model having the hospitalization – but not the patient - as the unit of analysis) have a particularly poor outcome. In addition, consideration of competing risks indicated little chance of biased results in our original model with miniscule parameter estimates in the competing risks model for all study covariates (Additional file 3).

## Discussion

To our knowledge, this is the most extensive examination of the association between blood culture testing and bloodstream infections on hospital mortality. We found that blood culture testing was common and that bloodstream infections were detected in almost 2% of hospitalizations. Patients undergoing blood culture testing were much sicker and had a notably higher risk of death in hospital that peaked around the time of their test. Even after adjusting for important confounders, patients with negative blood cultures still had a significantly increased risk of death in hospital. The risk of death was higher still in those with bloodstream infections with the risk being highest in bloodstream infections that: were detected in the first hospital day; were polymicrobial; occurred during a neutropenic episode or while exposed to immunosuppressants; or those due to *Clostridial* and *Candidal* organisms.

Our study has several important findings. First, we found that the independent risk of death in hospital increased significantly whenever blood cultures were ordered, even when those blood cultures did not grow a microorganism. This indicates that patients undergoing blood culture testing are sicker than others, even after adjusting for measured covariates that distinguished these patient groups (Table 1). Obviously, we do not believe that the actual act of measuring blood cultures increases the risk of death in hospital. Instead, we suspect that an increased risk of subsequent bad outcomes regardless of the blood cultures result is due to the test indicating a sicker population than is indicated by the measured covariates. We believe that this phenomenon is likely true for other tests (such as electrocardiogram, portable chest radiograph, cardiac enzymes, and arterial blood gases) that are done when patients deteriorate acutely. This observation should be considered when analyzing the influence of abnormal test results on hospital outcomes. Second, we found that the increased independent risk of death in hospital associated with negative blood cultures and bloodstream infections was maximal at the time that the blood culture was procured and decreased significantly over time. This likely reflects the benefit of treatments given to surviving patients. Finally, the harmful effect of bloodstream infections was highest with particular microorganisms (notably *Clostridial* and *Candidal*) and in immunocompromised hosts (those exposed to immunosuppressive agents and those with neutropenia). These results confirm how such patients with bloodstream infection must be treated aggressively.

Our study has several interesting comparisons with previous analyses of bloodstream infections and hospital mortality. The distribution of microorganisms that we identified in our cohort was very similar to that identified in previous analyses [2,5,12,13,15]. Similar to our results, several other analyses have found particularly high mortality rates in patients with *Candidal* bloodstream infection [5,6,14-16]. To our knowledge, ours is the most extensive analysis that included patients without blood cultures and those with negative blood cultures; this characteristic is necessary to precisely gauge the influence of bloodstream infection on mortality risk relative to other patients and independent of confounders associated with the actual measurement of blood cultures. Finally, several studies had previously found – in contrast to our study – that mortality risk was higher in those with nosocomial bloodstream infections [2,12,14,16]. We believe that these analyses are susceptible to time-dependent bias [26] since patients must remain alive in hospital for a specified period of time to be classified with nosocomial bloodstream infection. Since patients who remain in hospital longer tend to be sicker, such analyses could falsely attribute mortality risk from confounders in these patients to nosocomial bloodstream infection. Analyzing bloodstream infection as a time-dependent covariate within a survival model – as we did in this analysis – avoids this potential bias [25].

Our study has several notable attributes. We captured all hospitalizations, all blood cultures, and all bloodstream infections at our hospital during the study period. Our statistical model recognized the time-dependent nature of blood culture testing, bloodstream infections, and their association with death in hospital. However, several potential limitations of our study should be kept in mind. First, although we found that bloodstream infection was associated with an increased risk of death in hospital, we have no way of determining whether the infection actually *caused* the death. Primary data review would be needed to determine – if possible – whether or not the bloodstream infection caused a particular patient’s death. Such analyses are necessary to determine if and how we might intervene to improve outcomes with hospital-associated bloodstream infections. Second, our data did not account for treatment of bloodstream infections. Outcomes in patients admitted for community acquired pneumonia are improved significantly in those who receive antibiotics more quickly [27]. Berjohn et al. [11] found that patients with pneumococcal bacteremia receiving least 1 active antibiotic within 4 hours of blood cultures were significantly less likely to die in hospital (odds ratio [OR], 0.47; 95% confidence interval [CI], 0.2-1.0). It is possible that timeliness of appropriate antibiotics - and other interventions to control infection - could have an independent influence on death risk in all patients with bloodstream infections. Third, we did not have access to information about potential sources or causes of bloodstream infections, such as catheters. It is possible that mortality risk associated with bloodstream infections may change significantly based on the presence of foreign bodies. Fourth, the utilization of blood cultures requires a physician’s response to clinical data input and are not – by themselves – a pathophysiological marker. Physicians will vary in their response to various clinical data and, as such, will have different thresholds or likelihoods for ordering blood cultures. Therefore, the external validity of our findings to other centres could be questioned. However, supporting the generalizability of our findings is the large size and long duration of the study as well as its complete capture of all blood cultures at our large institution, all of which will ensure a large number of different physicians who were captured by the analysis.

## Conclusions

In summary, our study found that the risk of death in hospital is independently increased in patients with either negative blood cultures or bloodstream infections. In addition, death risk associated with bloodstream infections varied significantly by the patient’s immune status and the causative microorganism.

## Competing interests

The authors declare that they have no competing interests.

## Authors’ contributions

CvW conceived the study idea, conducted the analysis, and drafted the manuscript. JW created the analytical dataset and conducted the analysis. Both authors read and approved the final manuscript.

## Pre-publication history

The pre-publication history for this paper can be accessed here:

http://www.biomedcentral.com/1471-2334/14/36/prepub

## Supplementary Material

Additional file 1 Final model for the adjusted association of blood cultures and bloodstream infections with death in hospital. Click here for file

Additional file 2 **Comparison of parameter estimates in final model (Additional file **1**) having the admission or the patient as the unit of analysis.** Click here for file

Additional file 3 Competing risks analysis. Click here for file
