# Supplementary material for: Independent influence of negative blood cultures and bloodstream infections on in-hospital mortality
Source: BMC Infect Dis. 2014 Jan 21;14:36. doi: 10.1186/1471-2334-14-36 (PMC3917904; doi:10.1186/1471-2334-14-36)
Supplement: Additional file 1 — Final model for the adjusted association of blood cultures and bloodstream infections with death in hospital. [file 1471-2334-14-36-S1.doc]

**Additional file 1**: Final model for the adjusted association of blood cultures and bloodstream infections with death in hospital.

| **Factor** | **Parameter Estimate** | **Standard Error** | **Wald Chi-Square** | **P-value** | **Adjusted Hazard Ratio** | **-95% CI** | **+95% CI** |
| --- | --- | --- | --- | --- | --- | --- | --- |
| ***Non-interacting Covariates*** |  |  |  |  |  |  |  |
| Polymicrobial bloodstream infection | 0.47432 | 0.12684 | 13.9846 | 0.0002 | 1.607 | 1.253 | 2.06 |
| Bloodstream infection noted more than 24h after admission | -0.17372 | 0.06759 | 6.6053 | 0.0102 | 0.841 | 0.736 | 0.96 |
| Daily hospital death risk score | 0.83906 | 0.00837 | 10048.9151 | <.0001 | 2.314 | 2.277 | 2.352 |
| ***Interacting Covariates*** |  |  |  |  |  |  |  |
| Blood culture | 1.22972 | 0.09549 | 165.8457 | <.0001 | 3.42 | 2.836 | 4.124 |
| Quarter days since blood culture measured | 0.0000114 | 0.0005016 | 0.0005 | 0.9819 | 1 | 0.999 | 1.001 |
| Bloodstream Infection | 0.40725 | 0.05389 | 57.1009 | <.0001 | 1.503 | 1.352 | 1.67 |
| Neutropenic | -0.65379 | 0.18937 | 11.9193 | 0.0006 | 0.52 | 0.359 | 0.754 |
| Exposed to immunosuppressant | -0.24815 | 0.08384 | 8.761 | 0.0031 | 0.78 | 0.662 | 0.92 |
| Elixhauser score | 0.01949 | 0.00165 | 139.8044 | <.0001 | 1.02 | 1.016 | 1.023 |
| Emergent admission | 0.53951 | 0.07222 | 55.8005 | <.0001 | 1.715 | 1.489 | 1.976 |
| In intensive care unit | 0.76103 | 0.04485 | 287.9004 | <.0001 | 2.14 | 1.96 | 2.337 |
| ***Interactions*** |  |  |  |  |  |  |  |
| Square root(Quarter days since blood culture measured)*bloodstream infection | -0.05592 | 0.00872 | 41.0915 | <.0001 | 0.946 | 0.93 | 0.962 |
| Blood culture*Emergent admission | -0.65264 | 0.09087 | 51.5823 | <.0001 | 0.521 | 0.436 | 0.622 |
| Blood culture*Elixhause Score | -0.00807 | 0.00214 | 14.2384 | 0.0002 | 0.992 | 0.988 | 0.996 |
| Blood culture*Intensive care unit status | -0.6503 | 0.05035 | 166.8025 | <.0001 | 0.522 | 0.473 | 0.576 |
| Bloodstream Infection*Exposed to Immunosuppressant | 0.2922 | 0.07368 | 15.727 | <.0001 | 1.339 | 1.159 | 1.547 |
| Bloodstream Infection*Neutropenic | 0.58524 | 0.13985 | 17.5126 | <.0001 | 1.795 | 1.365 | 2.362 |
