# Supplementary material for: Independent influence of negative blood cultures and bloodstream infections on in-hospital mortality
Source: BMC Infect Dis. 2014 Jan 21;14:36. doi: 10.1186/1471-2334-14-36 (PMC3917904; doi:10.1186/1471-2334-14-36)
Supplement: Additional file 2 — Comparison of parameter estimates in final model (Additional file 1) having the admission or the patient as the unit of analysis. [file 1471-2334-14-36-S2.doc]

**Additional file 2:**  Comparison of parameter estimates in final model (Appendix A) having the admission or the patient as the unit of analysis.

|  | **Unit of analysis** | | |
| --- | --- | --- | --- |
|  | **Admission** | | **Person** |
| **Factor** | **Parameter Estimate** | **95% CI** | **Parameter Estimate** |
| *Non-interacting Covariates* |  |  |  |
| Polymicrobial bloodstream infection | 0.47432 | (0.2257, 0.7229) | 0.16624 |
| Bloodstream infection noted more than 24h after admission | -0.17372 | (-0.3062, -0.0412) | -0.19113 |
| Daily hospital death risk score | 0.83906 | (0.8227, 0.8555) | 0.84379 |
| Interacting Covariates |  |  |  |
| Blood culture | 1.22972 | (1.0426, 1.4169) | 1.30902 |
| Quarter days since blood culture measured | 0.0000114 | (-0.000971736, 0.000994536) | -0.0000000957 |
| Bloodstream Infection | 0.40725 | (0.3016, 0.5129) | 0.39578 |
| Neutropenic | -0.65379 | (-1.025, -0.2826) | -0.91481 |
| Exposed to immunosuppressant | -0.24815 | (-0.4125, -0.0838) | -0.27745 |
| Elixhauser score | 0.01949 | (0.0163, 0.0227) | 0.01794 |
| Emergent admission | 0.53951 | (0.398, 0.6811) | 0.4811 |
| In intensive care unit | 0.76103 | (0.6731, 0.8489) | 0.7346 |
| Interactions |  |  |  |
| Square root(Quarter days since blood culture measured)*bloodstream infection | -0.05592 | (-0.073, -0.0388) | -0.06265 |
| Blood culture*Emergent admission | -0.65264 | (-0.8307, -0.4745) | -0.73242 |
| Blood culture*Elixhauser Score | -0.00807 | (-0.0123, -0.0039) | -0.00835 |
| Blood culture*Intensive care unit status | -0.6503 | (-0.749, -0.5516) | -0.61353 |
| Bloodstream Infection*Exposed to Immunosuppressant | 0.2922 | (0.1478, 0.4366) | 0.29719 |
| Bloodstream Infection*Neutropenic | 0.58524 | (0.3111, 0.8593) | 0.73209 |
