# Supplementary material for: Independent influence of negative blood cultures and bloodstream infections on in-hospital mortality
Source: BMC Infect Dis. 2014 Jan 21;14:36. doi: 10.1186/1471-2334-14-36 (PMC3917904; doi:10.1186/1471-2334-14-36)
Supplement: Additional file 3 — Competing risks analysis. [file 1471-2334-14-36-S3.doc]

**Additional file 3:** Competing risks analysis

|  | **Outcome (censoring event)** | |
| --- | --- | --- |
|  | **Death in hospital**  **(discharge from hospital)** | **Discharge from hospital**  **(death in hospital)** |
| **Factor** | **Parameter Estimate**  **(95% CI)** | **Parameter Estimate**  **(95% CI)** |
| *Non-interacting Covariates* |  |  |
| Polymicrobial bloodstream infection | 0.47432 (0.2257, 0.7229) | -0.00259 (-0.0206416, 0.0154616) |
| Bloodstream infection noted more than 24h after admission | -0.17372 (-0.3062, -0.0412) | 0.000103 (-0.006463, 0.006669) |
| Daily hospital death risk score | 0.83906 (0.8227, 0.8555) | -0.0009153 (-0.001482328, -0.000348272) |
| Interacting Covariates |  |  |
| Blood culture | 1.22972 (1.0426, 1.4169) | 0.000497 (-0.0041678, 0.0051618) |
| Quarter days since blood culture measured | 0.0000114 (-0.00097, 0.00099) | -0.0000042126 (-0.0000263606, 0.0000179354) |
| Bloodstream Infection | 0.40725 (0.3016, 0.5129) | -0.0006256 (-0.0055256, 0.0042744) |
| Neutropenic | -0.65379 (-1.025, -0.2826) | 0.00251 (-0.0179916, 0.0230116) |
| Exposed to immunosuppressant | -0.24815 (-0.4125, -0.0838) | 0.0008222 (-0.0080566, 0.009701) |
| Elixhauser score | 0.01949 (0.0163, 0.0227) | 0.00000846795 (-0.00011422805, 0.00013116395) |
| Emergent admission | 0.53951 (0.398, 0.6811) | 0.0007981 (-0.0012599, 0.0028561) |
| In intensive care unit | 0.76103 (0.6731, 0.8489) | -0.00507 (-0.0122044, 0.0020644) |
| Interactions |  |  |
| Square root(Quarter days since blood culture measured)*bloodstream infection | -0.05592 (-0.073, -0.0388) | 0.0001926 (-0.000371292, 0.000756492) |
| Blood culture*Emergent admission | -0.65264 (-0.8307, -0.4745) | -0.00121 (-0.0055416, 0.0031216) |
| Blood culture*Elixhause Score | -0.00807 (-0.0123, -0.0039) | -0.000062 (-0.000239772, 0.000115772) |
| Blood culture*Intensive care unit status | -0.6503 (-0.749, -0.5516) | 0.00185 (-0.0061076, 0.0098076) |
| Bloodstream Infection*Exposed to Immunosuppressant | 0.2922 (0.1478, 0.4366) | -0.000844 (-0.009076, 0.007388) |
| Bloodstream Infection*Neutropenic | 0.58524 (0.3111, 0.8593) | -0.00232 (-0.0197836, 0.0151436) |
